# Important evidence of constant low CO_2_ windows and impacts on the non-closure of the greenhouse effect

**DOI:** 10.1038/s41598-019-41562-x

**Published:** 2019-03-22

**Authors:** Jing Zhao, Guoqing Li, Weihong Cui, Qianqian Cao, Haoping Zhang

**Affiliations:** 1 Key Laboratory of Digital Earth Science, Institute of Remote Sensing and Digital Earth, Chinese Academy of Sciences, Beijing, 100094 P. R. China; 2 University of Chinese Academy of Sciences, Beijing, 100049 P. R. China; 3 Hainan Key Laboratory of Earth Observation, Sanya, 572029 P. R. China; 4 National Engineering Center for Geoinformatics, Institute of Remote Sensing and Digital Earth, Chinese Academy of Sciences, Beijing, 100010 P. R. China; 5 International Eurasian Academy of Sciences (IEAS), Beijing, 100010 P. R. China; 6 China Centre for Resources Satellite Data and Application, Beijing, 100094 P. R. China

## Abstract

The CO_2_ distribution in the atmosphere remains unclear for the complexity of the long-range vertical transport process and other influencing factors. In this work, regression analysis was used to verify the accuracy of CO_2_ concentrations datasets. Geostatistical analyses were used to investigate the spatiotemporal distributions of CO_2_ at 7 levels from near the surface to the mid-troposphere (0~5 km). Spatial correlation and time series analyses were used to further determine the diffusion characteristics of the CO_2_ concentration based on the horizontal wind (NCEP R2), which is one of the main driving factors. The results showed that the horizontal, not vertical, diffusion of CO_2_ becomes increasingly more prominent with the decrease in atmospheric pressure to the mid-troposphere, whereas many regions, such as the Rocky Mountains and Qinghai-Tibet Plateau, have constant low values throughout the year due to the influence of high topography (up to 10.756 ppmv lower than that near the surface). These areas form low CO_2_ concentration ‘windows’ keeping letting thermal infrared energy out into space. This study is the first to question the existing view of the closure of the ‘greenhouse effect’. Future research studies should more precisely determine the closure threshold and the uncertainties about the surface fluxes.

## Introduction

Carbon dioxide (CO_2_), the concentration of which has increased by 40% since pre-industrial times, is the most significant human-emitted greenhouse gas responsible for global warming^1–3^. CO_2_ is a chemically inert gas^4^; thus, it can remain in the atmosphere and be transported for long distances. Surface fluxes, which add or subtract CO_2_, and the existing CO_2_ jointly determine the horizontal distribution and seasonal cycle of CO_2_ in the atmosphere^5^. This determination helps us examine the seasonal and annual evolutions of CO_2_ observations and better understand global climate change^6^. Numerous studies have focused on the spatial variation and transport processes of CO_2_. Due to the vertical transport of the CO_2_ cycle, the retrieved seasonal cycle from the Infrared Atmospheric Sounding Interferometer (IASI) data (which have a maximum sensitivity at 13 km) lags by 2 months compared to the surface and by 1 month compared to measurements from the Atmospheric Infrared Sounder (AIRS) (~11 km)^7^. Vertical wind motion via convection has been identified as the main driver of CO_2_ transport from the surface to the mid-troposphere over India^8^. In addition, the vertical transport of CO_2_ has been confirmed to vary by region and is mainly controlled by anthropogenic CO_2_ emissions and horizontal and omega winds^9^. Furthermore, in addition to satellite remote sensing data, data derived from aircraft and tethered balloons have been used to analyse the vertical distribution of CO_2_ and its characteristics^10–12^.

However, the long-range vertical transport process of atmospheric CO_2_ has not been fully investigated using observations from satellite sensors, especially based on Thermal Infrared (TIR)^9,13^, although the CO_2_ column abundance based on Shortwave Infrared (SWIR) and its retrieval algorithms have enhanced our knowledge of the global carbon cycle^14–17^. How this process operates in the mid-troposphere and near the surface from a global and regional perspective remains unclear due to several reasons. First, researchers lack high-precision observations of the vertical distribution of CO_2_ that cover long time periods (years) and different pressure layers^11^. Second, it is complex and difficult to simulate^18,19^ CO_2_ concentrations in the free troposphere using transport models because of the large model uncertainties^20,21^ and insufficient surface fluxes, which have not been entirely validated^22^. Furthermore, the vertical distribution of CO_2_ also diverges due to the different environmental characteristics of different regions, such as vegetation phenology, land composition, distribution of geographical features, atmospheric conditions, wind patterns, and anthropogenic emissions^16^. In addition, measurements performed by aircraft, stratospheric balloons, Light Detection and Ranging (LIDAR), tethered balloons, and high towers^23,24^ have insufficient vertical resolutions and dissatisfy the requirements at the maximum reachable sampling height around the world.

High spatio-temporal resolution satellite data have their advantages to fill this gap. Among all CO_2_ satellite observations, data from the Greenhouse Gases Observing Satellite (GOSAT) and AIRS can more precisely demonstrate a long-range vertical distribution of CO_2_, because of the different performance strengths of the two instruments. In this study, to determine the long-range distribution of CO_2_ at several pressure levels and better understand the closure of the atmospheric ‘window’^25^ through which heat from the Earth’s surface can radiate freely to space, we performed the following experiments and analyses. The consistency between near-surface CO_2_ concentrations (975 hPa) obtained from GOSAT and the surface fluxes achieved from World Data Centre for Greenhouse Gases (WDCGG) station data were verified. Two space-based observations of CO_2_ at the same level (500 hPa) from GOSAT and AIRS were compared to derive a higher precision data source of the CO_2_ distribution in the mid-troposphere. In addition, the spatiotemporal distribution of CO_2_ from the near-surface to the mid-troposphere and one main influencing factor (horizontal wind) were analysed to examine the global and regional (China and the United States) long-range transport effects on the CO_2_ concentration distribution. Finally, the relatively constant low concentrations of CO_2_ at the identified atmospheric window sites (Rocky Mountains and Tibetan Plateau) at various atmospheric levels are emphasized. This research provides the earliest evidence for the non-closure of the ‘greenhouse effect’, which is helpful for improving the public’s understanding of the dynamics of atmospheric CO_2_ driven by different variables^9^ and further confirms a new perspective of the ‘greenhouse effect’.

## Results

### Verification of the consistency of satellite-based and ground-based CO_2_ concentrations (975 hPa)

**Figure 1:**
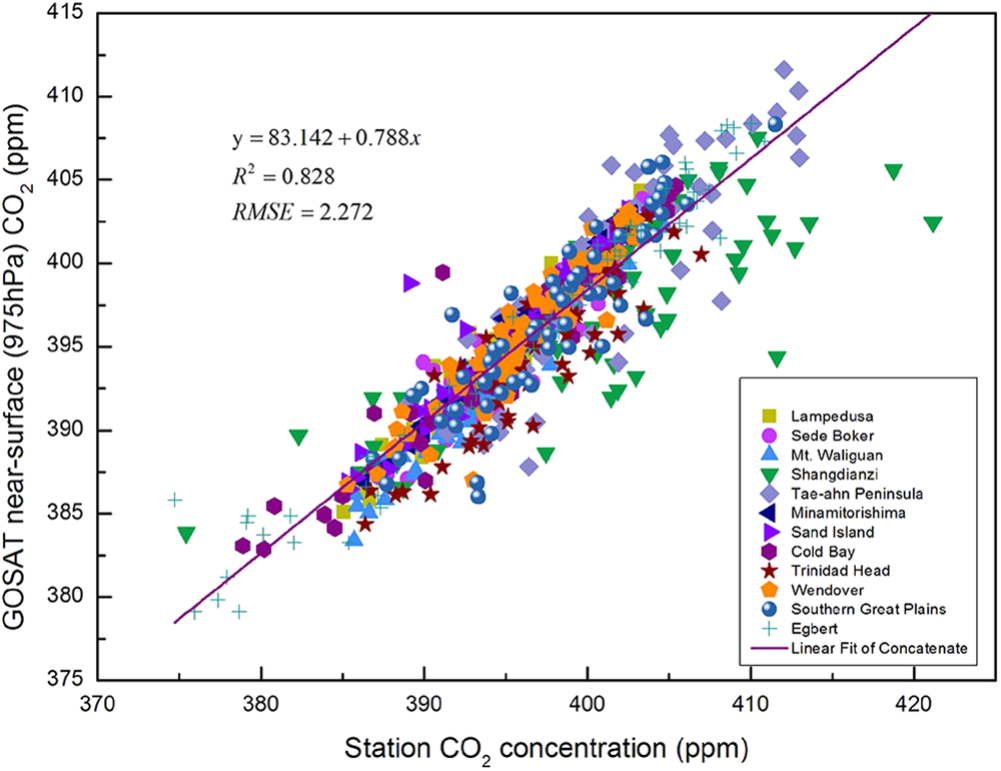
Regression between the near-surface (975 hPa) CO_2_ concentrations retrieved from GOSAT and 12 ground-based station observations from January 2010 to December 2014.

Fig. 1 shows the regression results from GOSAT and ground-based CO_2_ concentrations (975 hPa) at 12 ground stations from January, 2010 to December, 2014. There is a highly interdependent relationship between the two over the period (*β* = 0.788, *R*^2^ = 0.828, *RMSE* = 2.272). Table 1 displays an additional details about the statistical results. All of the correlation coefficients from the mid-latitude regions from 162.720°W to 75.570°E are greater than 0.8, and all exceed 0.89 except those from Shangdianzi. Second, the mean of the monthly average deviations is 1.828 ppmv, and all are less than 2 ppmv except for those from Shangdianzi, Cold Bay, and Wendover. The average deviation is less than 5 ppmv only in Wendover. Moreover, the mean annual rate of increase in the CO_2_ concentration from all stations is 1.708 ppmv/a, whereas the average increase from the GOSAT results is 1.794 ppmv/a. This difference is less than 0.086 ppmv/a.

**Table 1:** Statistics of the relationship between the near-surface (975 hPa) CO_2_ concentrations of GOSAT and WDCGG at 12 ground-based stations from January 2010 to December 2014.

Site Names	Ground−Based Station			Monthly Average (ppmv)			Yearly Growth (ppmv/a)			
	Latitude (°)	Longitude (°)	Altitude (m)	Ground	GOSAT	Bias	Ground	GOSAT	Bias	R
Lampedusa	35.520 N	12.630 E	45	394.927	394.884	0.043	1.675	1.81	−0.135	0.968
Sede Boker	31.120 N	34.870 E	400	396.15	395.514	0.636	1.803	2.029	−0.226	0.956
Mt. Waliguan	36.280 N	100.900 E	3810	394.614	393.311	1.303	1.737	1.728	0.009	0.979
Shangdianzi	40.650 N	117.117 E	287	400.574	396.897	3.677	1.823	1.748	0.075	0.816
Tae-ahn Peninsula	36.720 N	126.120 E	20	401.265	399.32	1.945	1.904	1.934	−0.03	0.857
Minamitorishima	24.280 N	153.980 E	7.1	395.072	395.295	−0.223	1.792	1.749	0.043	0.993
Sand Island	28.200 N	177.370 W	4	394.684	395.046	−0.362	1.683	1.711	−0.028	0.942
Cold Bay	55.200 N	162.720 W	25	398.494	394.951	3.543	2.05	1.875	0.175	0.957
Trinidad Head	41.050 N	124.150 W	107	397.272	395.351	1.921	1.51	1.773	−0.263	0.902
Wendover	39.880 N	113.720 W	1320	398.623	393.558	5.065	1.764	1.792	−0.028	0.93
Southern Great Plains	36.780 N	97.500 W	318	397.672	396.527	1.145	1.21	1.63	−0.42	0.896
Egbert	44.230 N	79.783 W	255	393.97	396.046	−2.076	1.548	1.743	−0.195	0.977
Absolute Mean	35.520 N	12.630 E	497	396.943	395.558	1.828	1.708	1.794	0.136	0.931

### Correlation analysis between CO_2_ concentrations of GOSAT and AIRS at 500 hPa

**Figure 2:**
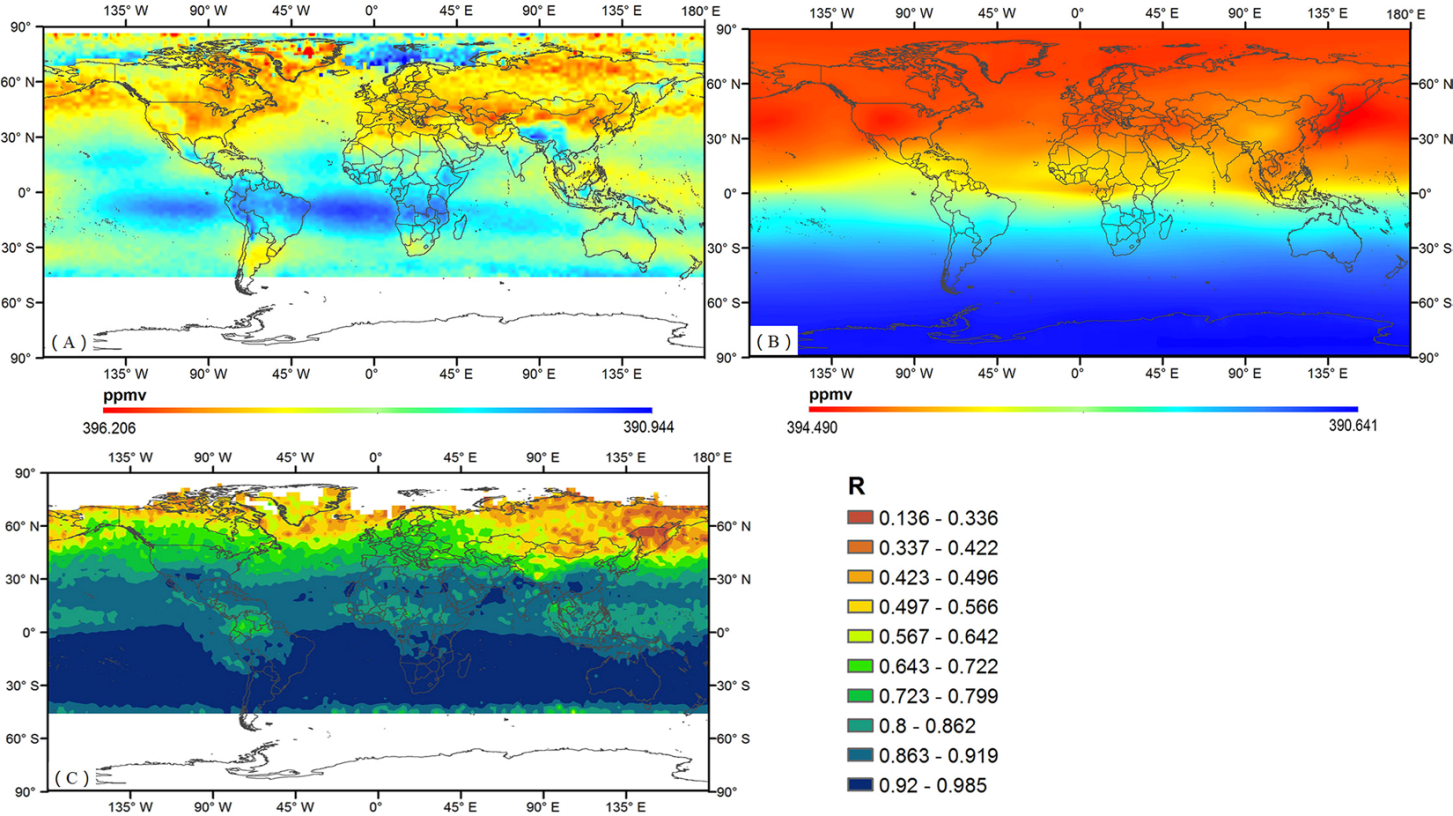
Yearly average CO_2_ concentrations and the correlation coefficient distributions of AIRS and GOSAT in the middle troposphere (500 hPa) from 2010 to 2014. (**A**) AIRS CO_2_. (**B**) GOSAT CO_2_. (**C**) Correlation coefficient.

The annual mean CO_2_ concentrations and the distribution of correlation coefficients between GOSAT and AIRS in the mid-troposphere (500 hPa) are shown in Fig. 2. The annual mean CO_2_ concentrations of the AIRS data range from 390.944 ppmv to 396.206 ppmv, while those from the GOSAT data range from 390.641 ppmv to 394.490 ppmv. The higher CO_2_ concentrations values at 500 hPa from these two satellites are mainly located in the Northern Hemisphere, whereas the lower values are located in the Southern Hemisphere. Significant differences are clear, although high- concentration areas with a zonal distribution can be found in both datasets. Fig. 2(B) reveals that the GOSAT CO_2_ concentration distribution is continuous with no spatial heterogeneity. In contrast, more details are apparent in the AIRS data in Fig. 2(A); higher-value areas extend over 30°~70° N and are concentrated mainly over the land masses. The correlation coefficient distribution between the two satellite products is displayed in Fig. 2(C). The correlation coefficients gradually increase from the Northern Hemisphere to the Southern Hemisphere.

Table 2 presents the statistical correlation results for the seasonal and annual CO_2_ concentrations at the chosen WDCGG stations, which quantitatively illustrate the differences between the two sets of satellite data. The annual increases, correlation coefficients, and seasonal fluctuations provide important information. Both the seasonal average and annual increases in CO_2_ concentrations for the two satellites reveal that the CO_2_ concentration in the mid-troposphere (500 hPa) has seasonal and monthly cycles. At almost all of the stations, the highest seasonal average CO_2_ concentration in the GOSAT data occurs in the spring, and the lowest occurs in the autumn (except for Shangdianzi), whereas in nearly half of the selected stations, the highest seasonal average CO_2_ concentration in the middle troposphere (500 hPa) in the AIRS data occurs during the winter. The measurements of the CO_2_ concentrations in the middle troposphere (500 hPa) from both satellites are weakly correlated with each other; the annual average and the 4 seasonal average values were 0.444, 0.167, −0.30, 0.306, and 0.436, respectively.

**Table 2:** Seasonal and annual CO_2_ concentration variations in the mid-troposphere (~500 hPa) from GOSAT and AIRS from 2010 to 2014 at the same locations as the WDCGG stations (Y.A. = Yearly Average; A.G. = Annual Growth).

Site Names	Y.A. (ppmv) (GOSAT)	Y.A. (ppmv) (AIRS)	A.G. (ppmv/year) (GOSAT)	A.G. (ppmv/a) (AIRS)	Bias				R	
					Y.A. (ppmv)		A.G. (ppmv/year)			
Lampedusa	394.168	395.100	2.159	2.055	−0.932		0.104		0.444	
Sede Boker	394.007	394.684	2.139	2.132	−0.677		0.007			
Mt. Waliguan	393.655	393.230	2.189	1.520	0.435		0.669			
Shangdianzi	393.935	395.445	2.126	1.922	−1.51		0.204			
Tae-ahn Peninsula	394.219	394.886	2.117	2.066	−0.667		0.051			
Minamitorishima	393.963	393.600	2.102	1.999	0.362		0.103			
Sand Island	394.193	393.694	2.128	1.967	0.499		0.161			
Cold Bay	394.239	395.055	2.128	1.720	−0.816		0.408			
Trinidad Head	394.209	394.716	2.138	1.958	−0.507		0.180			
Wendover	394.362	394.027	2.143	2.029	0.335		0.105			
Southern Great Plains	394.319	395.267	2.101	1.909	−0988		0.192			
Egbert	394.209	394.985	2.129	2.049	−0.776		0.08			
Site Names	**Seasonal Average (ppmv) (GOSAT)**				**Seasonal Average (ppmv) (AIRS)**				**R**	
	**Spring**	**Summer**	**Autumn**	**Winter**	**Spring**	**Summer**	**Autumn**	**Winter**	**Spring**	**Summer**
Lampedusa	401.453	396.866	395.786	401.344	400.923	399.608	397.971	400.375	0.167	−0.30
Sede Boker	400.930	397.123	395.841	400.564	398.403	399.086	397.791	399.207		
Mt. Waliguan	400.908	396.162	395.901	400.780	402.985	397.651	396.746	398.285		
Shangdianzi	401.628	394.818	396.050	400.630	400.226	400.127	397.566	400.485		
Tae-ahn Peninsula	401.558	395.916	392.309	401.300	399.036	400.082	398.264	397.817		
Minamitorishima	400.377	397.936	395.954	399.606	397.694	398.727	397.101	397.844	**Autumn**	**Winter**
Sand Island	401.040	397.675	396.023	400.378	398.687	398.410	396.602	398.173	0.306	0.436
Cold Bay	402.483	395.088	385.957	401.778	400.155	399.005	397.012	400.166		
Trinidad Head	401.799	397.067	395.781	400.601	399.357	398.958	398.112	399.516		
Wendover	401.657	397.510	359.869	400.730	398.376	399.573	396.983	397.731		
Southern Great Plains	401.483	397.211	395.909	400.724	399.476	399.466	398.851	399.643		
Egbert	401.988	395.866	396.080	401.385	399.509	399.171	399.447	399.289		

There are large differences between the two datasets, as discussed by *Christi et al*.^26^. The AIRS L3 data are based on cloud-free thermal infrared spectra and ideally map the distribution and transport of CO_2_ in the mid-troposphere. However, the GOSAT data, which use near-infrared bands that are more strongly influenced by aerosols, are less sensitive to CO_2_ in the mid-troposphere (500 hPa); this decrease in sensitivity causes the significant difference with the AIRS data as shown above. Because of the more detailed spatial heterogeneity and the smaller magnitude of the spatial variation, the CO_2_ concentration in the mid-troposphere (500 hPa) from AIRS is a better option for the next section.

### Spatiotemporal analysis of the global CO_2_ concentration from the near-surface to the mid-troposphere

**Figure 3:**
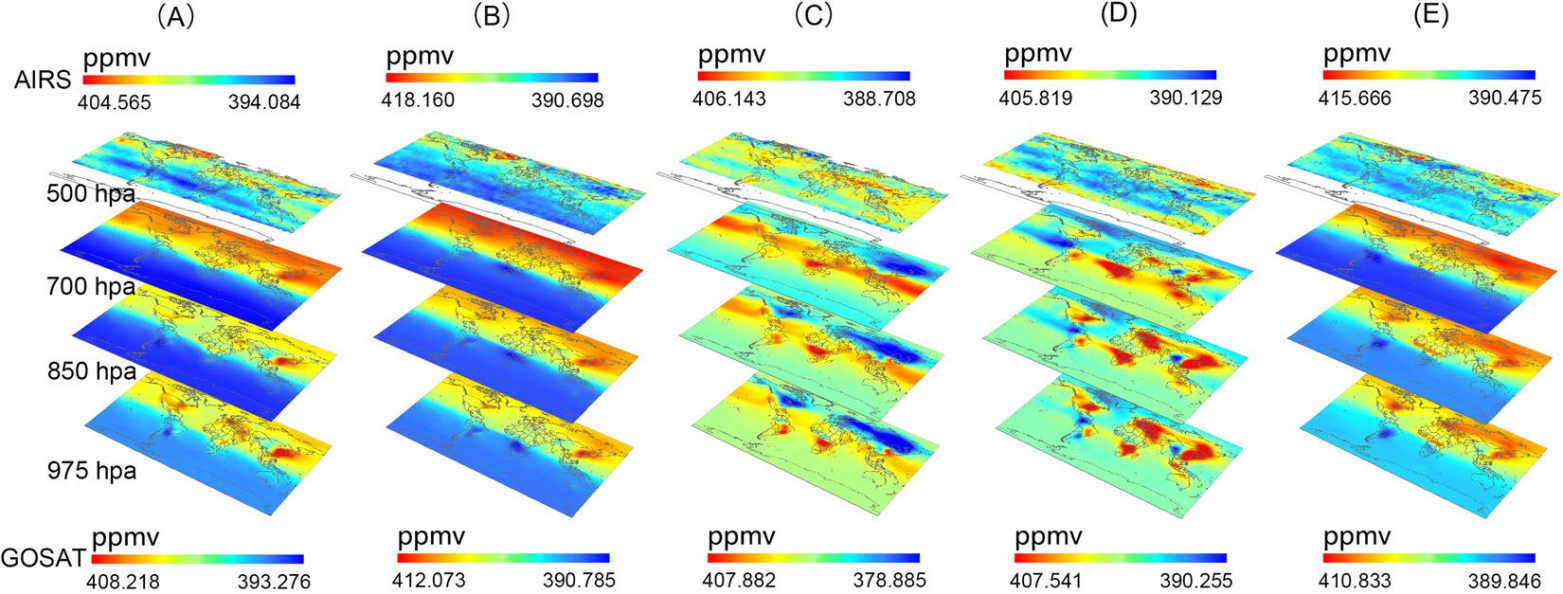
Spatiotemporal distributions of the global mean and seasonal average CO_2_ concentrations from the near-surface to the mid-troposphere (GOSAT was used at 975 hPa, 850 hPa and 700 hPa; AIRS was used at 500 hPa) from March 2014 to February 2015. (**A**) Yearly average. (**B**) From March to May. (**C**) From June to August. (**D**) From September to November. (**E**) From December to the following February.

The yearly average near-surface (975 hPa) CO_2_ concentrations exhibit significant spatial homogeneity at a global scale and heterogeneity at regional scales (Fig. 3(A)). The concentrations range from 393.276 ppmv to 408.218 ppmv. The concentrations are generally high in the Northern Hemisphere and low in the Southern Hemisphere, and there are small variations over the ocean, which illustrates the spatial homogeneity. However, there are significant differences between the Northern and Southern hemispheres and between the sea and land, which represents large heterogeneity. The higher concentrations converge in the mid-latitude regions (20°~55°N), such as eastern coastal China, coastal United States, France, Germany, Italy, Austria, the Czech Republic, Turkey, Syria, Iran, Iraq, Uganda, and Lake Victoria in Africa. This overall characteristic remains consistent from near the surface to 500 hPa in the mid-troposphere, where differences emerge. At this pressure level, globally, the yearly average CO_2_ concentration maintains the general pattern of being high in the Northern Hemisphere and low in the Southern Hemisphere, but narrows down from 394.084 ppmv to 404.565 ppmv. Clearly, the spatial homogeneity disappears, and the higher and lower values form horizontal striped distributions. The locations of the highest concentrations also move to the high-latitude areas (40°~80°N).

As shown in Fig. 3(B–E), the highest average concentration in the near-surface occurs during the first three months (March–May), whereas the lowest occurs during the second three months (June–August). The average CO_2_ concentration in the mid-troposphere has the same pattern. However, the specific seasonal distributions have differences. Near the surface, from March to May, the CO_2_ concentration generally has the same distribution as the yearly average, that is, higher concentrations in the Northern Hemisphere but lower concentrations in the Southern Hemisphere. The highest values are located in eastern coastal China, the east coast of the United States, and most of Russia, and the lowest are located in Brazil and southern Africa. In the second three months, nevertheless, high concentrations are spread almost entirely around the globe even though this is the low season. The concentrations vary evenly over the ocean but have large differences over the land. For example, almost all of the land masses have low concentrations except eastern coastal China because it is summer in the Northern Hemisphere, but remarkably high concentrations are located in Brazil and southern Africa in the Southern Hemisphere because it is a peak season of biomass burning. The discordance occurs from September to November. Almost all of the oceanic regions have uniformly lower concentrations, whereas nearly all of the land areas have higher concentrations, including eastern coastal China, the coastal United States, France, Germany, Italy, Austria, Czech Republic, Turkey, Syria, Iran and Iraq, Uganda, Lake Victoria in Africa, and Brazil, though, some land areas are different. South America (except Brazil) has lower concentrations. During the last three months period (December to the following February), the distribution is rather similar to that of the first three months (March - May), although there is no distinct zone of high concentrations in central Africa.

With increasing height, the seasonal variation in the spatial distribution of CO_2_ from the near-surface to the mid-troposphere shows noteworthy horizontal diffusion features in the same latitudinal bands. Up to the mid-troposphere, in addition to the horizontally striped distribution, there are additional regional variations. Several regions have continuously low CO_2_ concentrations for the entire year. These areas are located in three latitude zones. At low latitudes (between 20°S and 10°N), low CO_2_ concentrations are located over the Cordillera, Amazon Plain and Brazilian Plateau in South America and the Congo Basin, East Africa Plateau and Ethiopian highlands in central Africa, except for marine areas. In the middle latitudes (approximately 30°N), low CO_2_ concentrations cover the Rocky Mountains in the United States, the Arabian Plateau in the central Arabian Peninsula and the Qinghai-Tibet Plateau in China. In high-latitude areas, the low concentration areas are distributed from the East European Plain to the Chersky Ranges and around the Alaska Range.

### Typical regional horizontal transport (wind vector) driving CO_2_ in the near-surface and mid-troposphere

To further investigate the horizontal diffusion features and confirm the reasons for the continuous low CO_2_ regions in the mid-troposphere, we focus on China and the United States. These areas were chosen for three reasons. First, the sites of the WDCGG we used to verify the data consistency previously are in the middle latitudes of the Northern Hemisphere with high correlations. Second, China and the United States are both located within high-concentration areas, as shown in Fig. 3(A), and both are influenced by the monsoons. Third, regional heterogeneity of the CO_2_ concentrations can be found in both countries.

**Figure 4:**
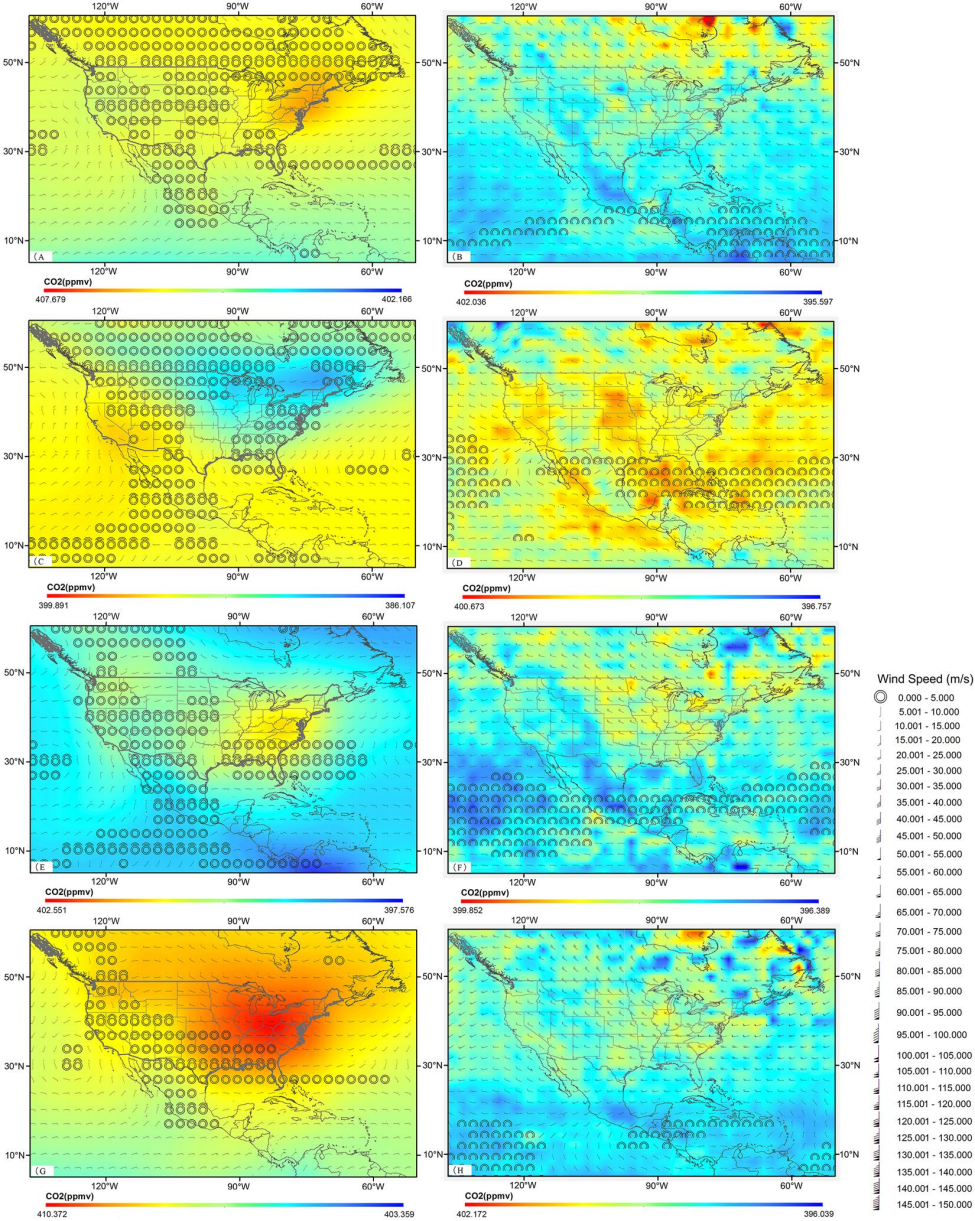
Seasonal average CO_2_ concentrations around the North America (Northern Hemisphere) driven by NCEP R2 horizontal wind from March 2014 to February 2015. (**A**) Spring (GOSAT (925 hPa)). (**B**) Spring (AIRS (500 hPa)). (**C**) Summer (GOSAT (925 hPa)). (**D**) Summer (AIRS (500 hPa)). (**E**) Autumn (GOSAT (925 hPa)). (**F**) Autumn (AIRS (500 hPa)). (**G**) Winter (GOSAT (925 hPa)). (**H**) Winter (AIRS (500 hPa)).

**Figure 5:**
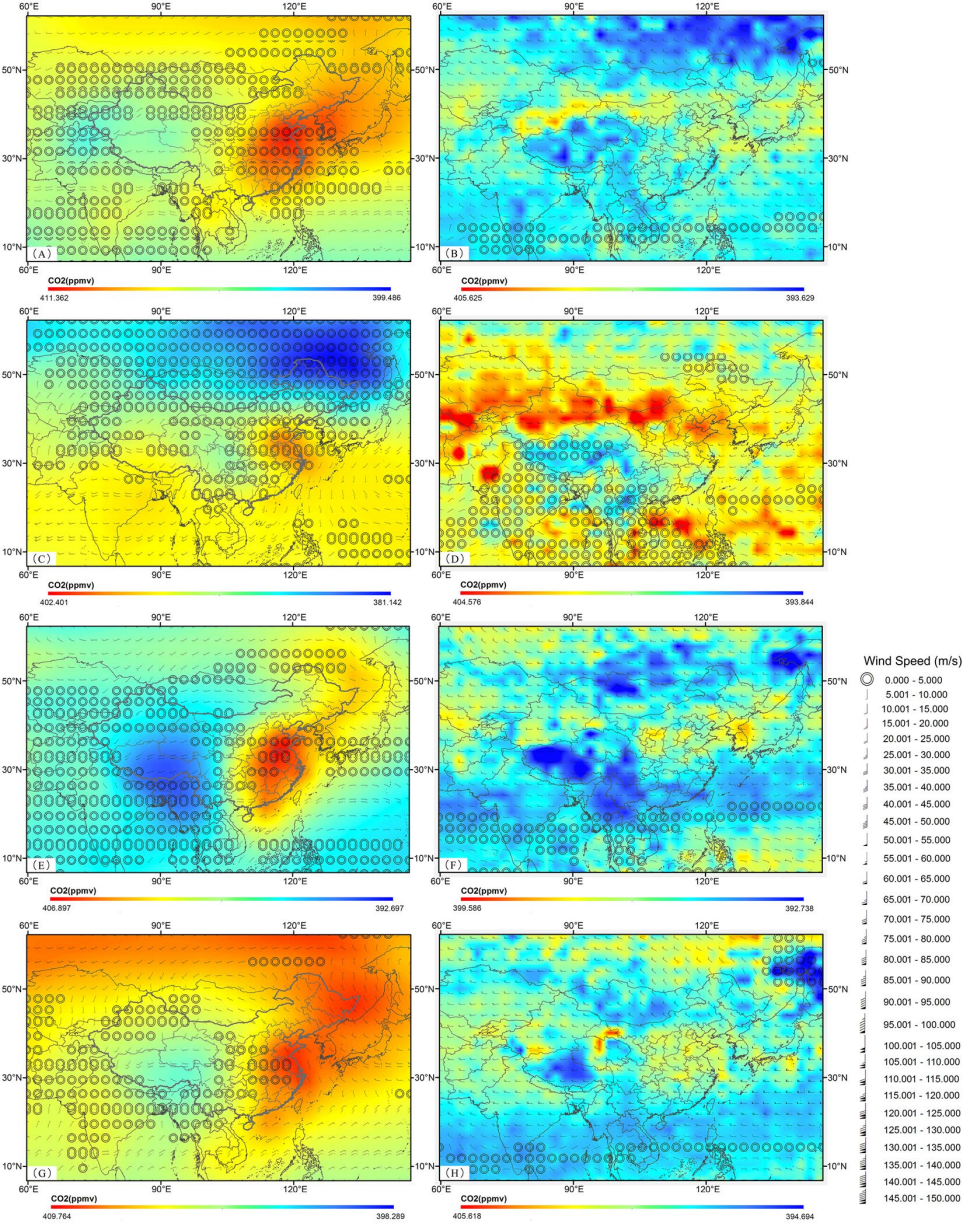
Seasonal average CO_2_ concentrations around the East Aisa (Northern Hemisphere) driven by NCEP R2 horizontal wind from March 2014 to February 2015. (**A**) Spring (GOSAT (925 hPa)). (**B**) Spring (AIRS (500 hPa)). (**C**) Summer (GOSAT (925 hPa)). (**D**) Summer (AIRS (500 hPa)). (**E**) Autumn (GOSAT (925 hPa)). (**F**) Autumn (AIRS (500 hPa)). (**G**) Winter (GOSAT (925 hPa)). (**H**) Winter (AIRS (500 hPa)).

As Figs 4 and 5 demonstrated, the near-surface CO_2_ concentrations are influenced by other factors other than the horizontal wind (Figs 4(A,C,E,G) and 5(A,C,E,G)). Up to the mid-troposphere, the concentrations are consistent with the horizontal wind vectors and topographic characteristics. In the summer, which is the most active plant growing season, the CO_2_ concentration in China is affected by the strong East Asian summer monsoon (southeast monsoon) and the Indian summer monsoon, which are caused by the different thermal properties between the land and sea. Due to the winds, a mass of CO_2_ is transported to the eastern coastal and southern regions of China, causing high CO_2_ concentrations (Fig. 5(C)). With increasing height, the horizontal wind becomes stronger, and the latitudinal horizontal diffusion feature becomes more obvious. Nevertheless, Fig. 5(D) displays that the summer plateau monsoon (PM) over the Tibetan Plateau results in a change in wind direction and low CO_2_ concentrations in this region, which is different to the near-surface region. Similarly, in the United States (Fig. 4(C)), the southeast monsoon and western wind have strong impacts on the CO_2_ concentration, causing the high concentrations along the east and west coasts. However, the Rocky Mountains maintain lower CO_2_ distributions, whereas almost all of the remainder of the United States is covered by higher CO_2_ concentrations due to the horizontal winds in the mid-troposphere (Fig. 4(D)). In the winter, northeastern China, where enormous amounts of natural and anthropogenic CO_2_ are released into the atmosphere, is seriously affected by westerlies, namely, the East Asian winter monsoon (Fig. 5(G)), and the high CO_2_ concentrations in northeast China move to the east coast. Northwesterlies and westerlies also severely impact the United States (Fig. 4(G)). The CO_2_ distribution in the southern United States is controlled by the west wind from the Atlantic, the northwest wind from the Arctic Ocean, and the south wind from the Gulf of Mexico, which lead to anticyclonic weather in the Arctic Ocean. Up to the mid-troposphere, due to the horizontal wind, the areas of high topography, such as the Tibetan Plateau, Altai Range, Mongolian Plateau and the Rocky Mountains, maintain continuously low CO_2_ distributions (Figs 4(H) and 5(H)). The spring and autumn both have relatively moderate weather conditions (Figs 4(A,B,E,F) and 5(A,B,E,F)), and the influences of the monsoons become weaker. As a result, the near-surface CO_2_ concentrations are affected more by the surface fluxes, whereas the CO_2_ distribution in the mid-troposphere has more obvious topographic characteristics.

What’s more, there are differences between the two countries. The Tibetan Plateau has a significant impact on the CO_2_ concentration in the mid-troposphere in China for the entire year, while the distribution in the United States is influenced less by the Rocky Mountains (Figs 4(B,D,F,H) and 5(B,D,F,H)) because of the much stronger effect of the west wind coming from the Atlantic, which is then altered by the northwest winds coming from the Arctic Ocean during the winter.

### Constant low CO_2_ concentrations in the Rocky Mountains and Tibetan Plateau

**Figure 6:**
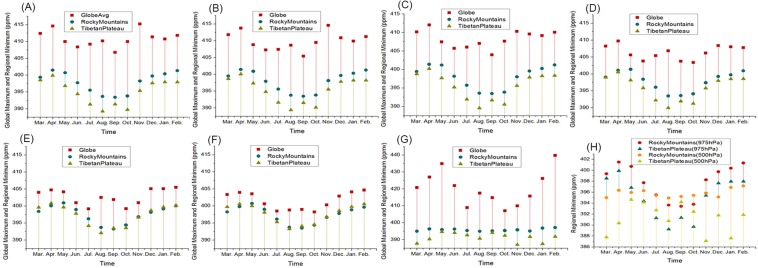
Variations in the monthly average CO_2_ concentrations from the near-surface to the mid-troposphere from March 2014 to February 2015. (**A**) GOSAT (975 hPa). (**B**) GOSAT (925 hPa). (**C**) GOSAT (900 hPa). (**D**) GOSAT (850 hPa). (**E**) GOSAT (700 hPa). (F) GOSAT (600 hPa). (**G**) AIRS (500 hPa). (**H**) GOSAT (975 hPa) and AIRS (500 hPa).

Previous studies^27–29^ have attempted to determine the global distribution of CO_2_ in the upper troposphere to the stratosphere (5~42 km). According to Mohamadou Diallo *et al*. (2017), at 50°~60° N latitude, although the mixing ratios from the upper troposphere to the middle stratosphere vary from relatively high to low, the CO_2_ concentrations decline substantially. In this study, the CO_2_ concentrations in the Rocky Mountains and the Qinghai-Tibet Plateau in China from the near-surface to the mid-troposphere (0~5 km) were obtained from validated satellite model simulations. Because there is no defined CO_2_ concentration threshold for the closure of the greenhouse effect, the low CO_2_ concentrations at the two high altitude sites, compared with the global average, were relied on to propose the existence of the atmospheric window (Fig. 6). As described previously, the global monthly average concentration decreases with increasing altitude (975~600 hPa) and to its minimum at 600 hPa; while it reaches much higher values in the mid-troposphere (500 hPa). The average CO_2_ concentrations in the Rocky Mountains and Tibetan Plateau are almost constant (from 390 ppmv to 400 ppmv) with increasing altitude (975~600 hPa) but fall noticeably to their minimum in the mid-troposphere (500 hPa). The CO_2_ concentrations at various atmospheric levels at the identified atmospheric window sites (Rocky Mountains and Tibetan Plateau) are consistently low. The seasonal variations in CO_2_ in the Rocky Mountains and Tibetan Plateau are exhibited in Fig. 6(H). Remarkably, from July to October, the monthly averages of the near-surface CO_2_ are much lower than those at 500 hPa. The most likely explanation for this phenomenon during the Northern Hemisphere wet summer (JJA months) is vegetation carbon sequestration, indicating that the constant low CO_2_ concentrations in the Rocky Mountains and Qinghai-Tibet Plateau are still affected by a variety of factors. Furthermore, it should be noted that new validated satellite data are needed to examine the average vertical CO_2_ profile above 500 hPa to confirm that the CO_2_ values remain low.

## Discussion

Prior studies have used the ground-based CO_2_ concentrations from WDCGG to validate satellite-retrieved CO_2_ data^19,30–32^ with high correlation coefficients. However, several studies have concluded that near-surface CO_2_ concentrations are overestimated in GOSAT XCO_2_ ^33^ data due to the uncertainties in both the satellite observations and model simulations^34,35^. In this work, the 11 correlation coefficients are greater than 0.89, and all but one deviation are less than 2 ppmv. This outcome suggests that the GOSAT inversion results from 30°~60°N can capture the seasonal CO_2_ variations^36^. Our results for the accuracy of the GOSAT near-surface CO_2_ concentrations provide foundational evidence for further discussion about the distribution and driving factors of CO_2_ and can potentially be representative of the surface fluxes. However, the uncertainties remain to be determined.

In previous published work, the largest negative biases in the Thermal and Near Infrared Sensor for Carbon Observation-Fourier Transform Spectrometer (TANSO-FTS) TIR V1 CO_2_ profile of GOSAT were found in the mid-troposphere region centred at 500–400 hPa^13^, and the monitoring accuracy of the GOSAT CO_2_ concentrations has been determined experimentally to be 4 ppmv^37,38^, which is less sensitive to CO_2_ in the mid-troposphere (500 hPa)^26^. The accuracy of AIRS is approximately 1–2 ppmv^36^. The CO_2_ concentration minimum in the mid-troposphere lags behind that in the near-surface region^39,40^, generally appearing after the autumn. This study found that the minimum GOSAT concentrations at 11 stations occur in the autumn, whereas approximately half of the AIRS minima occur in the winter, which is consistent with previous studies^9^. However, our results provide compelling evidence that the distribution of AIRS includes more details about the spatial heterogeneity. AIRS data thus provide greater potential for measuring the distribution of CO_2_ in the mid-troposphere and analysing the transport driven by the horizontal wind at this altitude.

Previous researchs have extensively investigated the CO_2_ concentration distributions in the near-surface region and the mid-troposphere^9,33^ and attempted to generate a global CO_2_ distribution with high accuracies and high spatiotemporal resolution using fused datasets and the gap-filling method^41^. Our team inspect the CO_2_ concentrations at 7 levels from the near-surface to the mid-troposphere (0–5 km) based on high spatial resolution satellite observations, which were derived via model simulations and recorded changes affected by surface fluxes and atmospheric transport. And we only presented 4 levels in Fig. 3 to improve the display of the quality of the figure to avoid a cluttered image and the omitted pressure levels don’t add value (not showing different distribution than the other used pressure levels). The results are different from those in other studies, almost all of which focus on regional characteristics^19^ or individual heights^13^. A recent published paper developed a new monthly zonal mean carbon dioxide (CO_2_) distribution from the upper troposphere to the stratosphere (5–42 km) over 2000–2010 based on a Lagrangian backward trajectory model driven by ERA-Interim reanalysis meteorology and tropospheric CO_2_ measurements^29^. The mixing ratios were discussed in detail. However, this study focuses on the overall distribution and local invariant features with increasing height to the mid-troposphere. As discussed above, the global spatial homogeneity and regional heterogeneity in the near-surface region transition to a horizontal diffusion band distribution with increasing height. However, with increasing altitude, the large topographic characteristics of the CO_2_ concentration become more obvious, even though they are influenced by the stronger horizontal wind. The Rocky Mountains in the United States and the Qinghai-Tibet Plateau in China have low CO_2_ concentrations for the entire year (Fig. 5). From March to June and from November to the following February, the CO_2_ concentrations in the mid-troposphere have even lower values, up to 10.756 ppmv lower than those near the surface. These large geographic features have large impacts on the CO_2_ concentrations in these two areas, resulting in persistent low CO_2_ concentrations. However, the other areas of low CO_2_ concentrations must be analysed further, and the factors that affect them require additional research.

These results provide a new perspective on the ‘greenhouse effect’. The ‘greenhouse effect’ is the process by which radiation from a planet’s atmosphere warms the planet’s surface to a temperature above what it would be without the atmosphere^42^. A real greenhouse works by reducing airflow so that warm air is kept inside^43,44^. CO_2_ is a strong absorber of thermal infrared energy with wavelengths longer than 12–13 micrometres, which means that increasing CO_2_ concentrations partially “close” the atmospheric ‘window’ through which heat radiated by the surface would escape to space^25^. But our results and the results from Mohamadou Diallo *et al*. (decreasing mixing ratios with increasing altitude from the upper troposphere to the middle stratosphere (~35 km))^29^ provide insight into the closure of the ‘greenhouse effect’; additional evidence for the ‘closure’ (when the ‘window’ closes) is needed or the ‘closure’ may not exist. The large topographic characteristics of the CO_2_ concentrations are not affected by the horizontal diffusion from the horizontal wind, and these regions with consistently low CO_2_ concentrations would absorb less radiation from the Earth’s surface, which would keep the atmospheric ‘window’ open, allowing more thermal infrared energy to escape into space. This scenario is analogous to the low-temperature holes in the Earth’s atmosphere, which should be called as ‘smoked ball effect’ globally and the ‘dome cloud effect’ regionally. Based on this discussion, we conclude that scientists who support the theory of the ‘closure’ of the ‘greenhouse effect’ should provide stronger and direct proof to demonstrate the circumstances under which it occurs (i.e., the closure threshold value). These areas of consistently low CO_2_ concentrations should not be ignored. They are encouraging and should be further explored and verified with other greenhouse gas data throughout the atmosphere.

Surface fluxes and long-range transport processes jointly influence the horizontal distribution of CO_2_ and its seasonal cycle in the atmosphere. This paper only focuses on the latter. The surface fluxes, including sources and sinks, are more important for the near-surface horizontal CO_2_ distribution when considering the horizontal wind. Nonetheless, this topic is too complicated to determine clearly. Although several studies have made contributions to this topic^19,30,45–48^, further work is needed to accurately quantify the sources and sinks with higher precision satellite data (e.g., OCO-2) and new data (e.g., TanSat) to ascertain the threshold for the closure of the ‘greenhouse effect’. Further study will also provide the potential to constrain the large uncertainties in the bio-flux in regional simulations during the summer^34,35^. Therefore, many uncertainties remain to be determined.

## Materials and Methods

### Satellite data

In January 23, 2009 GOSAT was launched and dedicated as the first satellite to detect the amounts of greenhouse gases (e.g., CO_2_ and CH_4_) in the atmosphere^45,49,50^. This study used the latest GOSAT dataset, the Level 4B (L4B) global CO_2_ distribution product (version 02.05) (https://data2.gosat.nies.go.jp/GosatDataArchiveService/usr/download/ProductPage/view). This product was available exclusively in the netCDF format and stored six-hourly global distributions of CO_2_ and CH_4_ from 2010 to 2015. These CO_2_ concentration values were derived via model simulations and depict changes in gas concentrations due to surface fluxes and atmospheric transport. The horizontal resolution of the concentration distribution data was 2.5 degrees, and the data were available at 17 vertical levels between the near-surface and the top of the atmosphere.

AIRS was the first of a new generation of high spectral resolution infrared sounder instruments included in the Aqua research mission^8^. It is located onboard the National Aeronautics and Space Administration (NASA) Aqua satellite and has a cross-track scanning grating spectrometer covering the spectral range from 3.74 μm to 15.4 μm with 2378 channels. The AIRS mid-tropospheric CO_2_ Level 3 Daily Gridded Retrieval product was used in this study (https://disc.gsfc.nasa.gov/datasets/AIRS3C2D_V005/summary)^51^. This product is a monthly gridded dataset with a 2.5 × 2 degrees (lon) x (lat) grid cell size, HDF4 format and was available from January 2010 to February 2017. The accuracy of the AIRS CO_2_ data was approximately 1–2 ppmv between 30°S and 80°N when compared to aircraft measurements and Fourier transform interferometers^36^.

GOSAT data include 17 vertical levels, while AIRS data only focus on the mid-troposphere. The aim of this study is to achieve an advantage-fused result of the CO_2_ concentration distribution from near-surface to mid-troposphere, which would benefit to draw a more accurate conclusion. AIRS data achieves a 2 ppmv accuracy in the tropics and mid-latitudes in the 15 micron band, associated Level 2 geophysical profiles of temperature, water vapor and ozone. The high accuracy of AIRS data (1–2 ppmv) is a big competitive advantage for mapping the distribution and transport of CO_2_ levels in the free troposphere. The detailed correlation analysis between the two data in the mid- troposphere can be found in the Results section.

### Ancillary data

WDCGG is one of the World Data Centers (WDCs) and falls under the Global Atmosphere Watch (GAW) programme, which is the lead programme for implementing recommendations of the Global Climate Observing System (GCOS) on essential climate variables (e.g., greenhouse gases, ozone, and aerosols). The GAW station network consists of 31 global and more than 400 regional stations with additional measurements from contributing stations. The aim of these observations is to obtain highly accurate local measurements. In recent years, the World Meteorological Organization (WMO) has adjusted its CO_2_ reference scale to WMOX 2007. The data from continuous CO_2_ measurements have been updated based on revised assigned values for the working standard cylinders supplied by National Oceanic and Atmospheric Administration’s (NOAA) Earth System Research Laboratory (ESRL)^47^. The data from continuous monthly mean CO_2_ measurements gathered from the 12 fixed stations listed in Table 1 are publicly available at the WMO WDCGG (https://gaw.kishou.go.jp/).

Monthly mean horizontal wind data from the National Centers for Environmental Prediction (NCEP) Department of Energy Reanalysis 2^52^ were used to analyse the horizontal transport of CO_2_ from the near-surface (975 hPa) to the mid-troposphere (500 hPa). The data were derived with a data assimilation technique using various *in situ* and satellite-based meteorological observations. The NCEP R2 has a global spatial coverage with a 2.5 degrees grid spacing and covers the period from January 1979 to December 2018. The monthly mean horizontal wind (zonal U- and meridional V-wind) data from NCEP R2 (between March 2014 and February 2015) were provided by the NOAA Earth System Research Laboratory’s Physical Sciences Division (PSD), located in Boulder, Colorado, United States, gathered from their website at https://www.esrl.noaa.gov/psd/, and downloaded from the data archive at http://www.esrl.noaa.gov/psd/data/gridded/data.ncep.reanalysis2.pressure.html#references.

### Methodology

**Figure 7:**
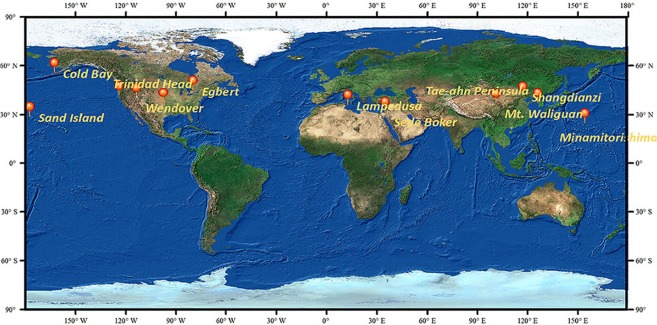
Geographical locations and spatial extent of stations chosen from the World Data Centre for Greenhouse Gases.

The sites from the WDCGG ground-based CO_2_ concentration data product chosen for this study are Lampedusa, Sede Boker, Mt. Waliguan, Shangdianzi, Tae-ahn Peninsula, Minamitorishima, Sand Island, Trinidad Head, Wendover, Southern Great Plains, and Egbert. They are distributed in the basemap imagery of ArcMap 10.5 for a more intuitive visualization (Fig. 7). The monthly means at each site, which were produced by averaging all valid measurements, including their uncertainties, are relative to standard air samples. The standard samples are detected using a Non-Dispersive Infra-Red (NDIR) sensor. The internal consistency of the working standards is +/− 0.02 ppmv (68% confidence interval)^53^. The WDCGG includes more than 430 stations, but we could choose only 12 stations because the continuous data from these sites include the same new long-period data as the satellite observations and cover the major human emission areas from 30°N to 60°N in the Northern Hemisphere. They are beneficial for satellite data verification for the following reasons. Almost all of the chosen stations are stable and provide measurements based on realistic scenarios without being directly affected by anthropogenic activities and local conditions. In addition, all of the data used here generally refer to average conditions, the long-term evolution of the atmospheric compositions and the chemical-physical properties, which also guarantees that the regional analysis error will be controlled and that the veracity will be assured.

The surface flux is one of the two main determinants that must be observed before evaluating the horizontal distribution and seasonal cycle of CO_2_ concentrations in the atmosphere. *Rayner et al*. demonstrated that a CO_2_ concentration accuracy lower than 1% (less than 4 ppmv) over an 8° × 10° grid could decrease the uncertainty of regional estimates of CO_2_ sources and sinks^54^. Although the accuracy of the CO_2_ concentrations for GOSAT is 4 ppmv, with the improvement in the algorithms^37^, the accuracy of the CO_2_ concentrations retrieved from GOSAT can reach ~1 ppmv^55^. In addition, *Takagi et al*. found that these higher precision CO_2_ products will lay a solid foundation for obtaining surface flux estimates^56^. We used regression analysis to estimate the relationship between the near-surface (975 hPa) satellite measurements and ground-based observations:

1 $$Y=\alpha +\beta X+\varepsilon ,$$

2 $${R}^{2}=1-\frac{S{S}_{res}}{S{S}_{tot}},$$

3 $$RMSE=\sqrt{\frac{{\sum }_{i=1}^{n}{({y}_{i}-\alpha -\beta {x}_{i})}^{2}}{n}},$$

where *X* and *Y* are two essential factors, *x*_*i*_ and *y*_*i*_ are the sample values, *SS*_*res*_ is the sum of squares for the regression, *SS*_*tot*_ is the total sum of squares, *R*^2^ is the coefficient of determination, and *RMSE* is the root-mean-square error. This was used to confirm whether the GOSAT near-surface CO_2_ measurements are reliable enough to represent the CO_2_ surface fluxes.

We used two satellite observations of CO_2_ concentrations in this study. Correlation analysis was applied to further analyse the reliability of the GOSAT near-surface observations and to ensure a better spatiotemporal distribution of CO_2_ in the mid-troposphere (~500 hPa) between AIRS and GOSAT:

4 $${R}_{xy}=\frac{{\sum }_{i=1}^{n}({x}_{i}-\bar{x})({y}_{i}-\bar{y})}{\sqrt{{\sum }_{i=1}^{n}({x}_{i}-\bar{x}{)}^{2}}\sqrt{{\sum }_{i=1}^{n}({y}_{i}-\bar{y}{)}^{2}}},$$

where $\bar{x}$ and $\bar{y}$ are the means of *X* and *Y*, respectively. Monthly average data were used to verify the two satellite-based products concurrently because only continuous monthly mean data are available from all of the selected ground-based stations and AIRS.

The long-range transport process is another key determinant when studying the CO_2_ concentrations at different heights. The direction and velocity of the prevailing winds were suggested to be the most important meteorological factors affecting near-surface CO_2_ concentrations^57^ and have important effects on the spatial distribution of near-surface CO_2_ ^19^. With increasing height to the mid-troposphere (~500 hPa), horizontal motion derived from convection or horizontal wind and dispersion (which changes according to the region) is regarded as the primary factor driving the horizontal transport of CO_2_ in the mid-troposphere^7,11,58^. The time-lag should also be considered. In this paper, we applied spatiotemporal geostatistics^59–61^ to map the CO_2_ concentrations at 7 levels from the near-surface to the mid-troposphere based on GOSAT and AIRS (500 hPa) observations, and the spatiotemporal distribution was analysed based on these concentrations. Spatial correlation analysis^62,63^ between the vector wind data and satellite observations at 925 hPa and 500 hPa in China and the United States was used to track the impact of the wind on the CO_2_ concentration distribution. Time series analysis was also used to analyse the variation in the CO_2_ concentration from the near-surface to the mid-troposphere.
